# Assessing the reliability of point mutation as data augmentation for deep learning with genomic data

**DOI:** 10.1186/s12859-024-05787-6

**Published:** 2024-04-30

**Authors:** Hyunjung Lee, Utku Ozbulak, Homin Park, Stephen Depuydt, Wesley De Neve, Joris Vankerschaver

**Affiliations:** 1 Center for Biosystems and Biotech Data Science, Ghent University Global Campus, Incheon, South Korea; 2 Department of Applied Mathematics, Computer Science and Statistics, Ghent University, Ghent, Belgium; 3 IDLab, Department of Electronics and Information Systems, Ghent University, Ghent, Belgium; 4 Korea University, Seoul, South Korea; 5 Erasmus Brussels University of Applied Sciences and Arts, Brussels, Belgium

**Keywords:** Data augmentation, Deep learning, Point mutations, Splicing, Translation initiation

## Abstract

**Background:**

Deep neural networks (DNNs) have the potential to revolutionize our understanding and treatment of genetic diseases. An inherent limitation of deep neural networks, however, is their high demand for data during training. To overcome this challenge, other fields, such as computer vision, use various data augmentation techniques to artificially increase the available training data for DNNs. Unfortunately, most data augmentation techniques used in other domains do not transfer well to genomic data.

**Results:**

Most genomic data possesses peculiar properties and data augmentations may significantly alter the intrinsic properties of the data. In this work, we propose a novel data augmentation technique for genomic data inspired by biology: point mutations. By employing point mutations as substitutes for codons, we demonstrate that our newly proposed data augmentation technique enhances the performance of DNNs across various genomic tasks that involve coding regions, such as translation initiation and splice site detection.

**Conclusion:**

Silent and missense mutations are found to positively influence effectiveness, while nonsense mutations and random mutations in non-coding regions generally lead to degradation. Overall, point mutation-based augmentations in genomic datasets present valuable opportunities for improving the accuracy and reliability of predictive models for DNA sequences.

**Supplementary Information:**

The online version contains supplementary material available at 10.1186/s12859-024-05787-6.

## Background

Deep Neural Networks (DNNs) have emerged as a popular and powerful tool in machine learning, revolutionizing the way we approach complex problems [1]. The early successes of DNNs in computer vision, for example in object recognition and image segmentation, demonstrated their ability to learn complex features from raw data and make accurate predictions [2]. These models have also shown great promise in the field of genomic data analysis, showcasing their ability to learn from and interpret large amounts of genetic data [3], and have seen increased use to solve a variety of biological problems including predicting translation initiation sites (TIS) [4–6], splice sites [7, 8], promoter sites [9], functional effects of non-coding variants [10], and to characterize protein-specific properties [11–13]

While DNNs have shown themselves to be powerful tools for predictive tasks, they also have several well-known shortcomings. One such shortcoming in utilizing DNNs is the requirement of data abundance, which can be a significant barrier to their use in areas where data availability is limited [14, 15]. A popular method to overcome this shortcoming is by using data augmentation techniques that have been widespread in both computer vision [16–18] and natural language processing [19–21]. In both of the aforementioned fields, data augmentation techniques not only improve the performance of models that are trained in a supervised fashion but also enable self-supervised learning, where the advent of self-supervised learning has given rise to now-famous frameworks like BERT [22], GPT [23], and LLaMA [24] in the domain of natural language processing, as well as MoCo [25, 26], DINO [27], and MAE [28] in computer vision.

Unfortunately, most data augmentation techniques used in computer vision or natural language processing cannot be directly applied to genomic data due to the unique characteristics of genomic datasets. Genomic data is highly structured and the application of even the smallest transformations may alter the properties of the underlying data or even introduce unintended signals. As a result, despite the tremendous impact of data augmentation techniques on the development of state-of-the-art AI-based solutions in other fields, the field of genomics has not yet been able to harness this powerful tool to its fullest extent.

Fortunately, genomic sequences come with certain properties that allow for other types of data augmentations, such as sequence flanking [29], base pair shifting [30], and sequence complementing [30, 31]. These data augmentations are reviewed in detail in “Related work” section and operate on the entire sequence (or part thereof), and often require specific conditions to be met. As a result, these augmentations have limited usefulness, or give rise to relatively few augmented sequences.

Unlike the aforementioned augmentation techniques, genetic mutations, as data augmentations, can be employed with any genomic data, and result in a plethora of new sequences. However, mutations as augmentations remain underutilized and underinvestigated, due to fears of introducing unintended signals into the data that would mislead the trained model. Indeed, almost by definition, mutations may exert an influence on the genomic sequence, fundamentally altering its inherent meaning. Nevertheless, we will show that the augmentation of training data through point mutations not only compensates for sporadic adverse impacts but also leads to a significant improvement in the performance of neural networks that are trained with it.

In this work, we investigate the usage of single nucleotide polymorphisms (i.e., point mutations) as a data augmentation method for genomic data. Our investigation focuses on translation initiation site (TIS) detection [5, 6], as well as splice site detection [7], since both are established as important tasks in genomics, cover both mutations in coding (i.e., exons) and non-coding (i.e., untranslated regions and introns) regions of genomic sequences, and are extremely sensitive to mutations [6]. Based on this investigation, we propose a principled and novel augmentation method that is straightforward to incorporate into any pipeline that employs such data. We find that the proposed augmentation method not only improves the performance of models but also helps models understand certain biological signals better. As a result of comprehensive experiments, we find that:

- Point mutations are useful in increasing the performance of neural networks across different genomic tasks when employed appropriately.
- Silent mutations (mutations which do not change the encoded amino acids) positively influence the performance of DNNs when applied moderately.
- Surprisingly, missense mutations (mutations which change the encoded amino acids) also lead to performance improvements and prove more useful than silent mutations in the majority of experiments.
- Nonsense mutations generally result in performance degradation in the majority of experiments.
- Similarly, random mutations in non-coding regions generally have a detrimental effect on performance.
- For all types of mutations, increasing the number of mutations leads to a significant decline in the model performance.

## Related work

In this section, we briefly cover the most commonly used data augmentation techniques for genomic data in conjunction with DNNs.

### Complement and reverse complement

DNA is composed of two complementary, anti-parallel strands [32]. This allows for the *reverse complement* to be used as a data augmentation technique [30, 31].

**Shortcoming** While the reverse complement is a useful genomic data augmentation method, it may not be biologically meaningful in certain situations. For coding proteins, the reverse complement sequence may not necessarily produce the same amino acid sequence as the original sequence due to the genetic code being read differently in the opposite direction. Another shortcoming is that the reverse complement method only provides a single additional sequence for each original sequence, leading to relatively few newly created data points.

### Flanking and shifting

[29] propose the usage of *data flanking* as augmentation where sequences are flanked with arbitrary subsequences while [30] use *sequence shifting* for data augmentation.

**Shortcoming** Flanking and shifting may not always be appropriate due to the unique structure and function of genomic data, thus heavily limiting the usage of this augmentation technique. DNA contains regional information, meaning that in a specific position, a certain nucleotide may be necessary for the proper function or binding of proteins [33]. For example, when base pairs are added or removed from a coding region, the reading frame of the sequence may be shifted, resulting in an incorrect translation of the genetic code. Similarly, shifting base pairs in the sequence may also introduce errors, resulting in the alteration or loss of important regulatory elements, such as promoters or enhancers [34].

### Masking

Masking is a data augmentation technique commonly used in computer vision and NLP. In computer vision, mask augmentation involves randomly masking out a portion of the input image, either by setting the pixel values to zero or by replacing the masked region with noise or other transformations. This forces the model to focus on the remaining unmasked regions of the image and to learn to recognize objects and patterns even when parts of the image are missing or occluded [35, 36]. In NLP, masking can be used to train models to predict missing words in a sentence or sequence of words. For example, in the Bidirectional Encoder Representations from Transformers (BERT) model, masking is used to randomly conceal some of the input tokens and then train the model to predict the original tokens based on the context [37]. This technique of masking can be utilized in genomic data augmentation as well [6, 38].

**Shortcoming** A masking operation does not generate novel sequences but rather alters the same sequence by concealing certain basepairs or regions. As a result, the utility of this approach is constrained because the scope of generated variations is limited [39].

### Codon degeneracy

[40] introduced a novel data augmentation technique that utilizes the inherent degeneracy of the genetic code. As such, it is one of the more recent approaches to employ and endorse the usage of mutations as augmentations. They observe that the inherent variability in the codon table of natural amino acids (using, for example, six codons for Serine and only one for Methionine) can introduce a bias in the learning process. To address this issue, they devise a method called Codon Balance, where three codons are allocated to each amino acid in a balanced manner. Furthermore, to evaluate the benefits of the natural codon relationship over an arbitrary one, they introduce the so-called Codon Shuffle approach, which randomizes the amino acid-to-codon relationship while preserving the original count of codons per amino acid.

**Shortcoming** Both Codon Shuffle and Codon Balance require additional calculations over the entire sequence to assess how the augmentation should be applied, potentially lengthening the training process, especially for models trained with longer sequences. Furthermore, since both approaches rely on the codon table of natural amino acids, the number of newly created sequences is limited (as their method avoids certain types of mutations). In contrast to their approach, our work investigates all point mutations—silent, missense, and nonsense in the coding region, as well as non-coding mutations—to address a broader question about the utility of mutations.

### Evolutionary mutations

Lee et al. [41] introduce a data augmentation that uses an evolutionary process (involving point mutations as well as larger structural variations) to increase genomic diversity while maintaining biological functionality. Their method consists of two steps: in the first, a deep neural network is trained on randomly mutated data, imparting some degree of robustness to the final model. In the second stage, the model is then fine-tuned on the original, unmutated data.

**Shortcoming** The process of repeatedly mutating the data and training the model on the augmented data increases the training time for the final, optimized model. This is especially the case for models trained on long sequences. Nevertheless, this method is conceptually the most similar to ours, the difference being that our training process is off-line, i.e. happens once, before training takes place.

**Table 1:** Characteristics of the datasets used in this study

Dataset	Objective	Task	Total	Positive seq.	Negative seq.	Pos./Neg. ratio	Source
CCDS	TIS	Train	728,990	27,834	701,156	0.0396	[42]
Chromosome21		Test	2,535,402	516	2,534,886	0.0002	
Gao15	TIS	Train	76,464	7148	69,316	0.1031	[4]
		Test	10,033	935	9098	0.1028	
NN269	Splice	Train	5788	1116	4672	0.2389	[43]
		Test	1089	208	881	0.2361	
Arabi Acceptor	Splice	Cross-val.	286,534	9309	277,225	0.0336	[44]
Arabi Donor	Splice	Cross-val.	272,715	9208	263,507	0.0349	[44]

## Methods

### Data

**Fig. 1:**
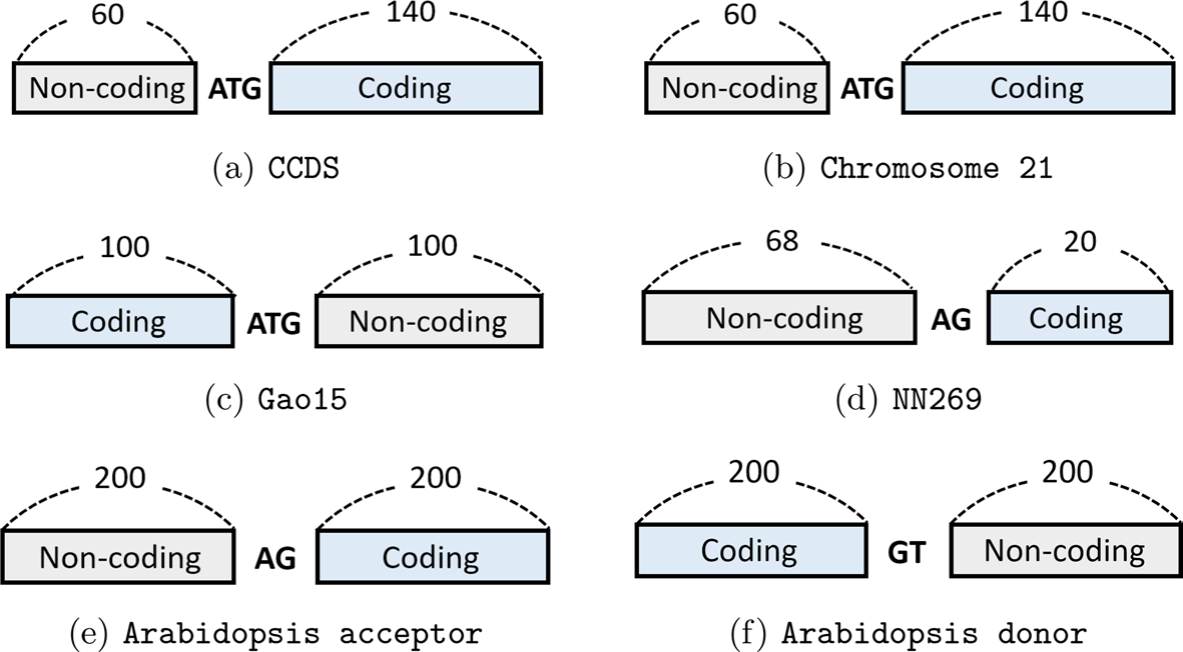
Arrangement of coding and non-coding regions in the six datasets used for TIS or splice site detection. Each sequence consists of either a TIS or a splice site, flanked by a coding or non-coding region of fixed length, as indicated in the figure

To facilitate comparisons with earlier research, we utilize datasets previously employed in relevant literature, particularly those that involve coding regions that are used for TIS and splice site detection since those tasks are more sensitive to point mutations compared to others. We make use of the CCDS, Chromosome-21, and Gao15 datasets for TIS detection [4, 5, 42] and the NN269 and Arabidopsis datasets for splice site detection [7, 43, 44]. In Table 1, we outline key characteristics of these datasets, and in Fig. 1, we provide a visual description of the sequences within each dataset.

- **CCDS and Chromosome-21** These datasets comprise pre-transcript human DNA sequences of 203 bp in length, each containing the canonical TIS ATG codon located at position 61 [42]. The label distribution in both datasets is heavily skewed, with a positive-to-negative ratio of 1/25 for the CCDS dataset and 1/4913 for the Chromosome-21 dataset.
- **Gao15** This dataset consists of DNA sequences that have a length of 203 bp, containing canonical TISs (ATG) positioned at 101 [45]. This dataset is extracted from QTI-seq data obtained from the HEK293 cell line along with the annotated TISs obtained from Ensembl v84 [46]. In total, the Gao15 dataset comprises 8083 positive samples and 78,414 negative samples derived from 4111 transcripts. We follow [4] in allocating 400 transcripts for testing purposes, while the remaining transcripts are considered as training data.
- **NN269** The NN269 dataset, which is a compilation of human splice sites obtained from 269 genes [47], comprises two separate datasets: donor splice sites and acceptor splice sites. However, we excluded the donor site sequences from our analysis because of their short length (15 bp) and focused solely on the acceptor dataset. The total length of sequences in this dataset is 90 bp, with the acceptor splice site AG located at position 69 [43, 48].
- **Arabidopsis dataset** The Arabidopsis dataset was curated for the purpose of predicting splice sites in *Arabidopsis thaliana*. It includes two datasets for acceptor and donor site detection, where each sequence in the datasets consists of 402 bp, with the splice acceptor site ‘AG’ and donor site ‘GT’ located at position 201 [44].

Note that as these datasets consist of short sequences with certain pre-defined genomic characteristics, they necessarily present a limited view of genomic diversity and come with certain inherent sources of bias. For example, the TIS datasets contain sequences with the canonical start codon ATG only, so that alternative translation initiation sites are not taken into account. A similar observation can be made for the splicing datasets, whose sequences are centered on the canonical splicing acceptor and donor sites AG and GT. Other sources of potential bias include the fact that the intronic and extronic part of the sequences are generally different in length (see Fig. 1) so that the latter carry more weight in the classification process. Lastly, all datasets were created based on the human genome and the *A. thaliana* genome. We emphasize, however, that our method is species-agnostic, and is therefore applicable to a wide range of genomic datasets.

### Deep neural networks

In recent years, deep neural networks (DNNs) have emerged as highly effective models for addressing sequence-related problems. Notably, several prominent models have been developed for DNN-based detection of translation initiation sites (TIS), including TITER [4], NeuroTIS [49], DeepTIS [50], TISRover [5], and TISRover+ [6]. Additionally, for splice site detection, Deep belief networks [51], Spliceator [52], SpliceRover [7], and SpliceAI [8] were created. TISRover and SpliceRover are highly specialized convolutional neural networks tailored for TIS and splice site detection, respectively. For our experiments, we utilize TISRover and SpliceRover for a number of reasons. Their performance approaches that of the state-of-the-art for the given task, and due to their moderate complexity, they permit an uncomplicated analysis for biological explainability (as done in “Discussion” section). Furthermore, as both TISRover and SpliceRover are convolutional neural networks, they exemplify an architecture widely applicable in genomic deep learning, suggesting that findings derived from these methods are applicable to deep neural networks for other tasks as well.

### Error measurements

Commonly used metrics like accuracy can present a misleading picture when evaluating neural networks on genomic data, due to the pronounced class imbalance between positive and negative samples. Instead of accuracy, we use a number of performance metrics that are suitable for imbalanced data, as described below.

- **fpr80** In order to mitigate potential misleading results in datasets with label skewness, we employ the false-positive rate at a fixed sensitivity of 0.8 (fpr80) as the benchmarking metric for the Chromosome-21 dataset, following the proposal by [42].
- **auROC** The area under the Receiver Operating Characteristic (auROC) curve is used as the evaluation metric for the NN269 and Gao15 datasets, as it is particularly suitable for heavily skewed data due to its robustness in handling imbalanced class distributions.
- **auPRC** The performance evaluation for the Gao15 dataset involves two metrics: auROC and the area under the Precision-Recall Curve (auPRC). The auPRC specifically addresses the challenge of skewed data by considering the precision-recall trade-off.
- **Pr95** In the evaluation of the Arabidopsis acceptor and Arabidopsis donor datasets, the precision (Pr) for sensitivity or recall of 0.95 is employed as a performance measure. The original authors of SpliceMachine [44] defined this metric to assess the effectiveness of the models.

These metrics were chosen because they were introduced with the datasets introduced in “Data” section, to evaluate the efficacy of machine learning models trained on their respective datasets. Note that for fpr80, lower is better, while for the other metrics (auROC, auPRC, Pr95), a higher value is better.

### Point mutations

Point mutations are changes in a single nucleotide base pair of a DNA molecule. They can occur spontaneously during DNA replication, or they can be induced by environmental factors such as radiation or chemicals. Point mutations can result in various types of alterations in the genetic code, including silent, missense, and nonsense mutations.

- **Silent** A silent mutation is a type of point mutation that does not change the amino acid sequence of the protein that is being encoded.
- **Missense** Missense mutations involve a change in a single DNA base pair that leads to the incorporation of a different amino acid in the protein sequence.
- **Nonsense** Nonsense mutations are mutations that introduce a premature stop codon in the DNA sequence. As a result, protein synthesis is prematurely terminated, giving rise to truncated proteins that frequently exhibit nonfunctional characteristics.
- **Non-coding** Mutations that take place in regions of the genome other than the coding region, such as in introns or in the 5’ UTR, are referred to as non-coding mutations. These mutations can have detrimental effects on protein delivery and timing of production, protein localization, and protein abundance as a whole [53].

The advantage of using point mutations as a data augmentation method is that it yields a method that is generally applicable to sequence data (i.e. not limited to a specific genomic task) and sufficiently powerful to result in significant performance improvements. Our method is not the first to employ point mutations (see [40, 41] for other mutation-based approaches) but it is the first to compare the performance improvements stemming from different mutation types.

## Results

**Fig. 2:**
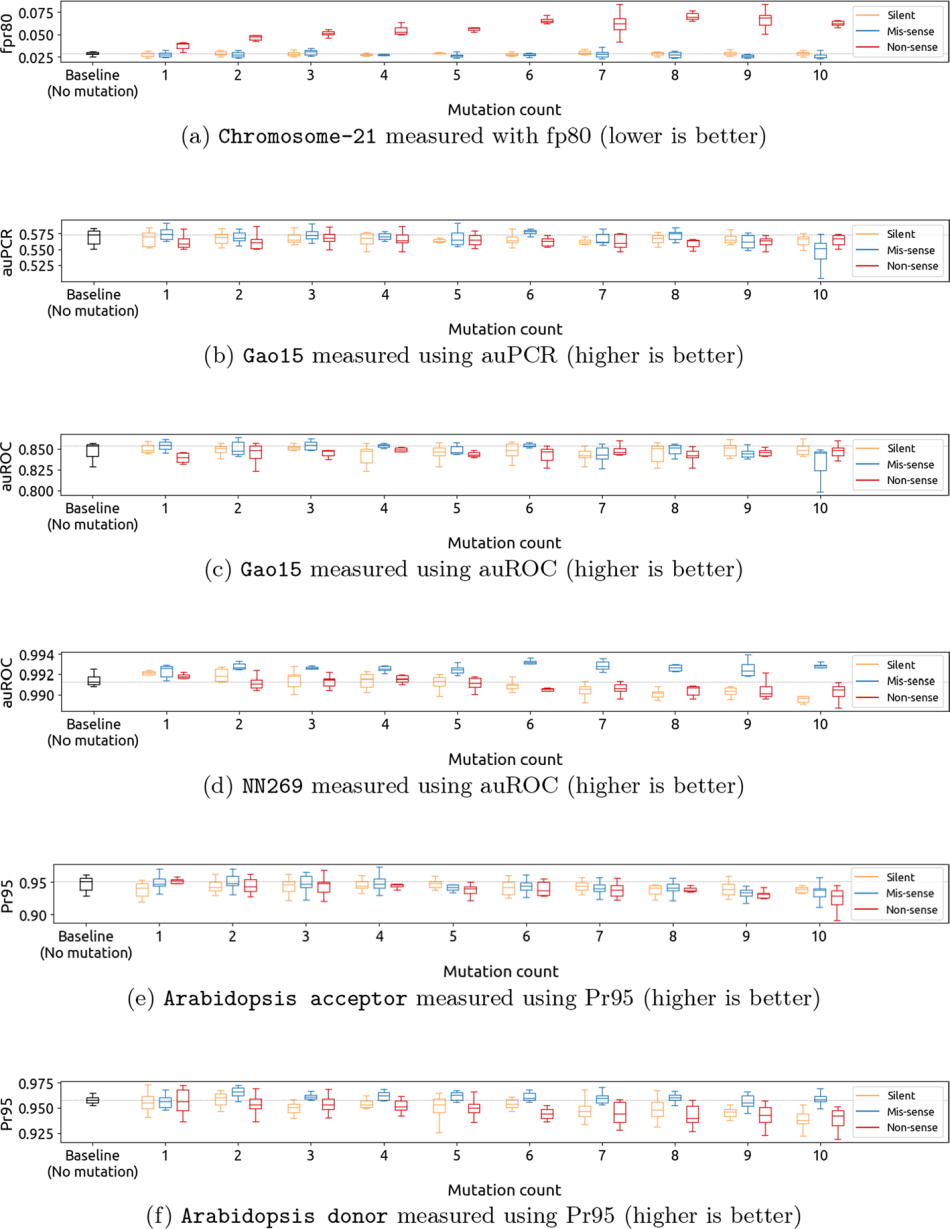
Changes in neural network performance for each of the five datasets after introducing up to 10 point mutations of different types in the coding region. Applying a moderate number of silent and missense mutations improves the performance, while large numbers of missense and nonsense mutations are generally detrimental

To investigate the effects of mutations on the genomic sequences, we conduct a number of experiments where we introduce up to 10 point mutations for each mutation type, including silent, missense, nonsense mutations, and random mutations in the non-coding region. In each run, we apply only one type of mutation, maintaining consistency throughout the experimentation process, and train and evaluate the model on the augmented data (using the evaluation metrics described in “Error measurements” section). Each run is repeated six times, with different random seeds.

**Fig. 3:**
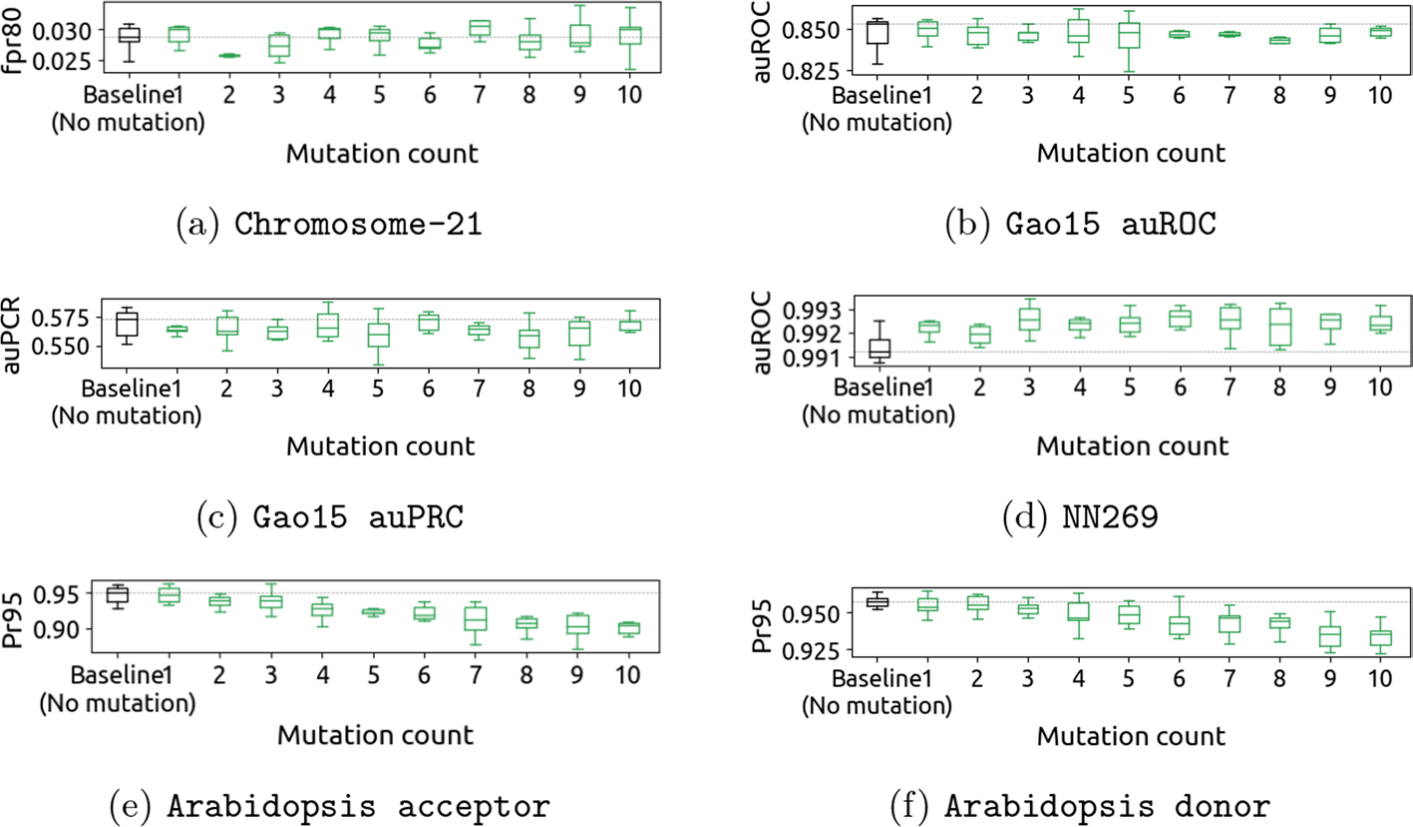
Changes in neural network performance for each of the five datasets after introducing up to 10 random mutations in the non-coding region. Applying random mutations generally has a detrimental effect on performance

To conduct a comparative analysis, we contrast the performance of the baseline approach, which does not involve any mutations, with the sequences that have undergone mutations. Figures 2 and 3 show for each dataset the distribution of evaluation metrics across training runs, as a function of the mutation count. Tables 2, 3, and 4 list the maximum accuracy across training runs for each mutation type.

Based on the aforementioned results, the primary observation we make is that excessive numbers of mutations adversely affects model performance across various mutation types, resulting in a decline in evaluation metrics. In contrast, a moderate level of augmentation (up to three point mutations) generally has a positive impact on model performance.

**Fig. 4:**
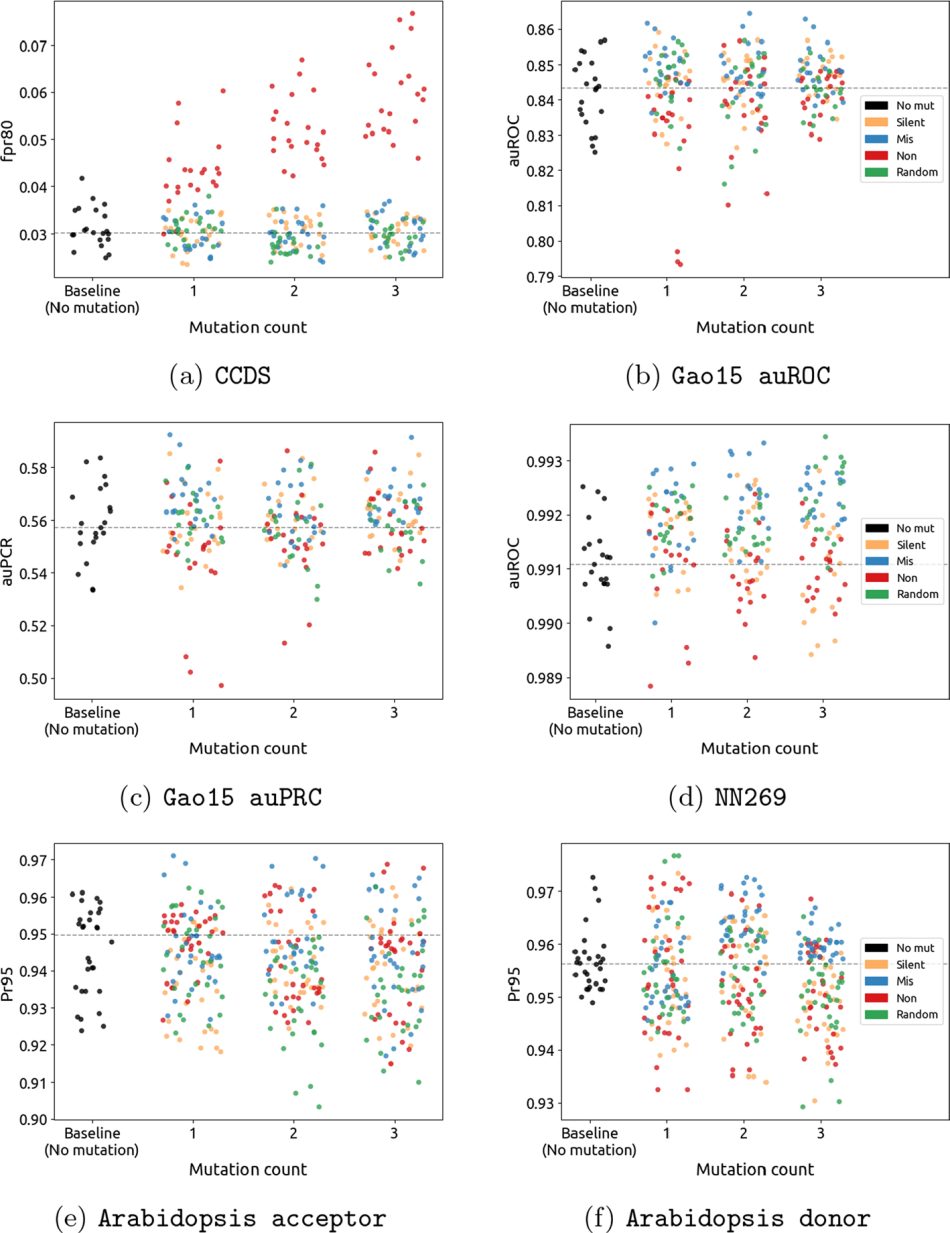
Effect of applying different mutation types for each dataset under comparison. Up to three mutations are applied, since higher mutation counts generally have a detrimental effect. The vertical dashed line indicates the median accuracy for the baseline case, in which no mutations are applied. Dots in the scatter plot indicate repetitions of the same experiment, with a different random seed, as explained in the body of the text

To further understand the effect of a moderate number of mutations, we show in Fig. 4 the accuracy of individual training runs, separated by mutation type and for one, two and three mutations. Specifically, for each training run, we display the three best outcomes of training in order to account for the stochastic variability of accuracy during training. These outcomes are compared with the mean performance of training runs without any augmentation, indicated by the black horizontal line. A more detailed view is provided by Additional file 1: Figs. S1, S2, and S3, which show the distribution of error metrics for each dataset and mutation type separately. Additional file 1: Tables S1 and S2 furthermore provide the results of a Mann–Whitney-U test at the 5% significance level, comparing each mutation type/count with the baseline, where no mutations are applied.

When it comes to mutation types, we observe that nonsense mutations are generally detrimental to model performance, resulting in a significant increase in the error. This is especially visible for Chromosome 21, which shows significant increases in the fpr80 metric (see Fig. 4 and Additional file 1: Fig. S1(a)), indicating a decrease in accuracy. This observation conforms with our expectations, and we further discuss the reasons behind this observation in “Nonsense mutations” section. On the other hand, we expected silent mutations to be mostly harmless since this type of mutation does not affect the encoded amino acid. However, our experiments show otherwise, with at best no improvement. Similar to other types of mutations, silent mutations also adversely affect model performance when they are applied in abundance (usually when the mutation count is larger than three). Further discussions on this topic can be found in “Silent mutations” section. Lastly, missense and random mutations in the non-coding region, which we believed would not lead to substantial improvements, show surprising levels of performance improvements, which are significant on all datasets except Chromosome 21 and Arabidopsis Acceptor. This is further discussed in “Missense mutations” and “Random mutations” sections, respectively.

**Table 2:** Effect of *silent mutations* as an augmentation technique across datasets and performance metrics

Dataset	Metric	Baseline	Mutation count									
			1	2	3	4	5	6	7	8	9	10
Chrom21	$\downarrow$ fpr80	.025	**.023**	**.025**	**.025**	.026	**.025**	.026	.027	**.025**	.026	**.025**
Gao15	$\uparrow$ auROC	.857	**.859**	**.857**	**.857**	**.857**	**.858**	**.859**	.854	**.858**	**.862**	**.863**
	$\uparrow$ auPRC	.584	**.585**	.583	**.585**	.578	.576	.582	.570	.578	.582	.576
NN269	$\uparrow$ auROC	.993	.992	**.993**	**.993**	.992	.992	.992	.991	.991	.991	.991
A.Acc	$\uparrow$ Pr95	.961	.953	**.962**	**.962**	.961	.960	.960	.957	.946	.959	.945
A.Don	$\uparrow$ Pr95	.973	**.973**	.967	.958	.962	.965	.961	.968	.968	.959	.953

Highlighted values indicate improvements over the baseline, where no mutations are applied

**Table 3:** Effect of *missense mutations* as an augmentation technique across datasets and performance metrics

Dataset	Metric	Baseline	Mutation count									
			1	2	3	4	5	6	7	8	9	10
Chrom21	$\downarrow$ fpr80	**.025**	**.025**	**.024**	**.025**	**.022**	**.023**	**.024**	**.023**	**.024**	**.024**	**.023**
Gao15	$\uparrow$ auROC	.857	**.862**	**.864**	**.863**	**.857**	**.858**	**.858**	.856	.856	.855	.849
	$\uparrow$ auPRC	.584	**.593**	.583	**.592**	.579	**.592**	.582	.583	**.585**	.576	.575
NN269	$\uparrow$ auROC	.993	**.993**	**.993**	**.993**	**.993**	**.993**	**.994**	**.994**	**.994**	**.994**	**.993**
A.Acc	$\uparrow$ Pr95	.961	**.971**	**.970**	**.966**	**.974**	**.968**	**.962**	.957	.956	.944	.957
A.Don	$\uparrow$ Pr95	.973	.968	**.973**	.967	.969	.967	.968	.971	.966	.966	.969

Highlighted values indicate improvements over the baseline, where no mutations are applied

**Table 4:** Effect of *non-sense mutations* as an augmentation technique across datasets and performance metrics

Dataset	Metric	Baseline	Mutation count									
			1	2	3	4	5	6	7	8	9	10
Chrom21	$\downarrow$ fpr80	.025	.030	.042	.046	.050	.052	.057	.042	.065	.051	.058
Gao15	$\uparrow$ auROC	.857	.846	**.857**	.848	**.859**	.849	.854	**.860**	.853	.852	**.860**
	$\uparrow$ auPRC	.584	.582	**.586**	**.586**	**.587**	.580	.572	.576	.566	.572	.574
NN269	$\uparrow$ auROC	.993	.992	.992	.992	.992	.992	.991	.991	.991	.992	.991
A.Acc	$\uparrow$ Pr95	.961	.958	**.963**	**.969**	.957	.950	.955	.957	.950	.946	.945
A.Don	$\uparrow$ Pr95	.973	**.973**	.970	.969	.961	.968	.952	.958	.958	.957	.951

Highlighted values indicate improvements over the baseline, where no mutations are applied

## Discussion

In this section, we discuss the implications and findings of our study, analyzing the results and focusing on their biological significance and relevance. We subdivide our discussion into four parts, with each part focusing on a different mutation type.

### Silent mutations

      **Observation** We observe that for the NN269 dataset, the application of silent mutations in moderate numbers (usually less than 3) results in enhancements in performance, as can be seen in Table 2 and Fig. 4. Figure 2 shows that a higher number of point mutations application of point mutations (more than three) often impairs the performance of the splicing site detection process, as it potentially disrupts the accurate identification and recognition of splice sites.

**Biological significance** Silent mutations, which preserve the amino acid sequence of a protein, can still influence protein expression and abundance. Based on this information, we hypothesize that the application of silent mutations would be a suitable candidate as a data augmentation for genomic data.

Somewhat unexpectedly, we observe that the usage of silent mutations as augmentation in large amounts negatively impacts the performance of the model. We believe this is due to their potential impact on the biochemical activity or functional properties of the protein itself [54]. While previously synonymous codon mutations (i.e., silent mutations) were considered to have no effect, multiple research efforts (see [55] and the references therein) have shown that codon usage affects protein structure and gene expression through effects on co-translational protein folding, translation efficiency and accuracy, mRNA stability, and transcription.

### Missense mutations

      **Observation** In addition to silent mutations, the moderate application of missense mutations can significantly enhance performance. Notably, Table 3 exhibits a majority of cases where missense mutations result in model improvements, indicating a more pronounced impact achieved through the usage of missense mutations. Figure 4 likewise shows a majority of cases in which performance improves upon the application of missense mutations, compared to silent mutations. Remarkably, among the various mutation types examined, there were no cases of performance deterioration under the moderate application of missense mutations.

**Biological significance** Unlike silent mutations, missense mutations can have various potentially negative effects, ranging from altering macromolecular stability to perturbing interactions and cellular localization. These mutations may disrupt protein stability, hydrogen bonds, dynamics, and activity, ultimately leading to the onset of diseases [56]. Initially, we expected missense mutations to have a negative impact on the model performance. However, experimental results show the opposite, as we observed missense mutations to be the most beneficial augmentation technique across all datasets. This revelation leads us to believe that the models are relatively robust to mutations of this type when it comes to evaluated tasks (TIS detection and splicing). Upon closer examination, the possibility of amino acids mutating into other similar amino acids via missense mutations, particularly taking into their polar or hydrophobic properties, may explain the observed effect. In such cases, although the encoded amino acid differs, its properties may still bear similarity to those of its unmutated counterpart, thereby potentially reinforcing the training signal for the model.

### Nonsense mutations

      **Observation** Figures 2 and 4 shows that applying nonsense mutations as an augmentation method results in a decrease in performance compared to the baseline approach. This decrease is particularly noticeable in datasets focused on TIS detection, such as the Chromosome-21 and Gao15 datasets, highlighting the disruptive effect of nonsense mutations on translation initiation. As shown in Fig. 2, we also observe a decrease in performance when applying nonsense mutations for splice site detection. Although there were no cases where nonsense mutations improved the performance, the impact on splice site detection is not as significant as for TIS detection.

**Biological significance** Nonsense mutations, which can potentially prematurely terminate translation, have significant implications in the final stage of mRNA translation. Accurate termination is crucial for proper protein synthesis and maintaining cellular proteomes, with release factors playing a vital role in identifying stop codons [57]. Premature termination can lead to the accumulation of truncated and potentially harmful proteins. Additionally, unstable mRNA can result in translational errors, triggering nonsense-mediated mRNA decay (NMD), a specialized mechanism for rapid degradation of faulty mRNAs [58].

Nonsense mutations also have a profound impact on the splicing process, leading to nonsense-associated alternative splicing, as explained by the scanning and splice motif disruption models. The splice motif disruption model suggests that nonsense mutations disrupt ESEs, also mentioned in “Silent mutations” section. Genome-wide transcriptomic and k-mer enrichment analyses support this model, demonstrating that ESEs are prone to disruptive nonsense mutations due to their purine-rich composition and the scarcity of termination codons. Additionally, both in-frame and out-of-frame mutations to premature termination codons (PTCs) are associated with exon skipping. These findings emphasize the importance of considering splice motif modulation in comprehending the etiology of diseases associated with PTCs [59]. This highlights the complex interplay between nonsense mutations and splicing processes.

The impact of applying nonsense mutations as an augmentation method varies across different TIS datasets. In the Chromosome-21 dataset, the performance is substantially affected, as indicated by a much higher fpr80 (Fig. 2a). In contrast, the Gao15 dataset also shows a decrease in performance with nonsense mutations, but the effect is less pronounced. We believe that the dissimilarity in impact can be attributed to the distinct curation of data in these datasets. The Gao15 negative dataset was constructed by selecting up to 10 codon sites of the same triplet within the same transcript as negative samples for each TIS in the positive dataset, taking into account the leaky scanning nature of the translation initiation process [4]. However, this specific approach was not employed when creating the Chromosome-21 dataset. This dataset was constructed with 294 genes and sequences with consensus TIS (i.e. ATG) were selected as positive data, while the remaining ATGs were included as negative data. The difference in data curation likely contributes to the varying degrees of impact observed with nonsense mutations in these datasets.

### Random mutations

      **Observation** Random mutations in non-coding regions generally have a detrimental effect on performance, as evidenced by Fig. 3. Also, as the number of random mutations applied increases, the performance further deteriorates, indicating a strong correlation between the extent of mutation and the decline in performance. However, for the NN269 dataset, although a small improvement is observed, this improvement is exceedingly marginal compared to the overall performance degradation caused by random mutations.

**Biological significance** Mutations occurring in non-coding regions have been shown to have an impact on the problem of TIS detection. Recent studies have highlighted the functional role of somatic non-coding variants, particularly in the context of transcriptional and post-transcriptional gene regulation [60]. Additionally, mutations in the 5’ UTR have been implicated in disease pathogenesis, as alterations in the translation initiation consensus sequence can lead to context-dependent leaky scanning and initiation from downstream ATG codons. For instance, mutations in the 5’ UTR of the BRCA1 gene have been found to affect translation efficiency and contribute to breast cancer aggressiveness [61]. Moreover, 5’ UTR mutations have the potential to disturb the anticipated secondary structure and resultant inaccessibility of the cap structure can inhibit translation [62].

Mutations occurring within introns may have a profound impact on splicing, resulting in the generation of aberrant transcripts and contributing to the development of various diseases. As with point mutations in the coding region, mutations in introns can disrupt existing splice sites or splicing regulatory sequences (intronic splicing silencers, enhancers, and snoRNAs) [63, 64]. These mutations disrupt proper intron recognition, leading to errors during the splicing process and alterations in the open reading frame [65, 66]. Consequently, splicing mutations can directly cause disease or influence disease susceptibility and severity. For instance, a single point mutation within the first intron of the beta-globin gene can cause beta thalassemia [6]. Thus, the interplay between splicing efficiency and intron removal is critical for maintaining proper gene expression and functionality.

## Implementation

In order to foster reproducible research and to enable the straightforward usage of the proposed augmentation method, we are sharing an easy-to-use implementation of the method in Python. In what follows, we provide details about the usage of this implementation and discuss its limitations.

The implementation contains a single class called AugmentMutations(mut_type, orf_pos, mutable_bp_range, unmutable_bp_range). This class can be initialized with the desired mutation type that will be employed: silent, missense, nonsense, or random and the position of the open reading frame (ORF) of the sequence (0, 1, or 2). Furthermore, the implementation is flexible enough to accommodate multi-range mutation locations, which are handled by the next two parameters: mutable_bp_range and unmutable_bp_range, both of which take lists of lists containing bp ranges. The primary reason behind the implementation of unmutable_bp_range is to prevent mutations on regions of the sequence that should be conserved (such as the translation initiation or the acceptor/donor sites). The implementation can perform a given amount of mutations within the ranges provided in mutable_bp_range, based on the mutation type and the ORF. As a result, this implementation can be easily used in any pipeline as an additional data augmentation with very little additional effort. An example usage of the implementation is provided in Listing 1. Usage of the implementation for mutation augmentation used in this work.
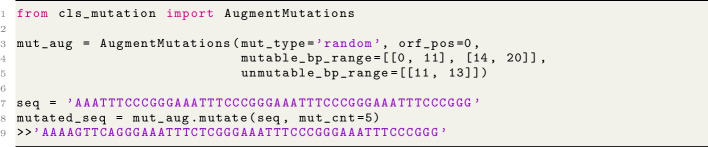


Although the proposed method and its implementation are flexible to be used in a wide-range of scenarios, the primary shortcoming in augmenting silent, missense, and nonsense mutations is the necessity of the ORF location. Indeed, if the ORF location is not known for the sequence, every mutation essentially becomes a random mutation. This is the fundamental limitation of using mutations as augmentations in coding regions where the ORF is not known. As such, in such scenarios, we advise the reader to exercise caution while using the proposed method.

## Conclusions

In this study, we focused on exploring the utility of various mutation types as augmentation methods for genomic datasets. Through a series of comprehensive experiments, we investigated the impact of silent, missense, nonsense, and random mutations on classification problems using genomic data, particularly TIS detection and splicing. To the best of our knowledge, this is one of the first large-scale computational experiments of a generally applicable data augmentation method specifically designed for genomic data.

We found that silent mutations, while preserving the amino acid sequence of a protein, positively influenced protein expression and abundance, and resulted in small but significant performance improvements, making them a viable option for enhancing performance when applied in moderate numbers. Similarly, the strategic application of missense mutations led to improvements in performance. Although missense mutations have various effects on protein function, they exhibited a similar impact to silent mutations on splicing processes. On the other hand, applying nonsense mutations as an augmentation method generally resulted in performance degradation, particularly affecting datasets focused on TIS detection. Nonsense mutations, which prematurely terminate translation, can lead to the accumulation of truncated and potentially harmful proteins and have complex effects on splicing processes. Furthermore, random mutations in non-coding regions consistently had a detrimental effect on performance, disrupting intron recognition and proper splicing processes.

We expect that our data augmentation technique will be most useful in the context of building DNNs for which there is a limited amount of data available. In this case, data augmentation serves a dual purpose of increasing the size of the underlying dataset, and helping to elucidate the biological function, as demonstrated in “Discussion” section.

The findings from this study highlight the potential benefits of employing strategic silent and missense mutations as augmentation methods for genomic datasets, while also underscoring the importance of understanding the impact of different mutations on splicing processes. Exploring the use of different mutations as augmentation methods in genomic datasets provides valuable opportunities for improving the accuracy and performance of TIS and splice site detection. It also provides valuable insights into the optimization of augmentation strategies, suggesting the importance of carefully selecting the appropriate level and type of augmentation to enhance the accuracy and reliability of predictive models for DNA sequences. We expect that our method will, with the same level of tuning, be able to deliver similar performance improvements for other genomic tasks as well.

For future work, we are interested not just in the improvement of deep learning models per se, but also in the degree of biological explainability that augmentations provide for a well-trained model. It is clear, after all, that a data augmentation method must balance creating new sequences with the need to (approximately) preserve the biological function(s) in those sequences, and as we have argued in “Discussion” section and elsewhere [6], deep neural networks learn biological features precisely through their susceptibility to point mutations. Furthermore, we are also interested in quantifying the improvements made in the feature-space of models using various interpretability techniques similar to the works of [68]. We plan to extend this line of enquiry towards other tasks in genomic machine learning, especially in gene expression regulation.

An additional avenue for investigation involves applying the methodology to alternative types of data that involve proteins such as [69–71]. The findings of this study demonstrate that incorporating point mutations in genomic data can enhance the robustness of a deep learning model and provide insights into biological functions. It is anticipated that a mutation-based data augmentation method for protein data, for example, could play a comparable role.

### Supplementary Information

** Additional file 1.** Supplementary figures and tables.

## Data Availability

The datasets and code used in this paper can be downloaded from https://zenodo.org/doi/10.5281/zenodo.10457889.
