# Supplementary material for: Assessing the reliability of point mutation as data augmentation for deep learning with genomic data
Source: BMC Bioinformatics. 2024 Apr 30;25:170. doi: 10.1186/s12859-024-05787-6 (PMC11059627; doi:10.1186/s12859-024-05787-6)
Supplement: Supplementary file 1 — Additional file 1. Supplementary figures and tables. [file 12859_2024_5787_MOESM1_ESM.pdf]

Supplementary Materials for: Assessing the Reliability of Point Mutation as Data Augmentation for Deep Learning with Genomic Data

Hyunjung Lee[4,†], Utku Ozbulak[1,†], Homin Park[1,3], Stephen Depuydt[5], Wesley De Neve[1,3], Joris Vankerschaver[1,2]

[1]Center for Biosystems and Biotech Data Science, Ghent University Global Campus, Incheon, South Korea.
[2]Department of Applied Mathematics, Computer Science and Statistics, Ghent University, Ghent, Belgium.
[3]IDLab, Department of Electronics and Information Systems, Ghent University, Ghent, Belgium.
[4]Korea University, Seoul, South Korea, Belgium.
[5]Erasmus Brussels University of Applied Sciences and Arts, Brussels Belgium.

Corresponding author: joris.vankerschaver@ghent.ac.kr;
†These authors contributed equally to this work.

1 Deep Neural Networks and Training

In the main text of our study, we use TISRover and SpliceRover, Deep Neural Networks (DNNs) optimized for detecting Translation Initiation Sites (TIS) and splice sites, respectively. We provide a detailed descriptions of both models in Figure 1 where a ReLU activation is positioned after each trainable layer.

In order to train models, we mostly follow the work of [1, 2] but incorporate recent advances in DNN training such as (1) a single epoch warm-up training where the learning rate starts from zero and is linearly increased up the initial learning rate and

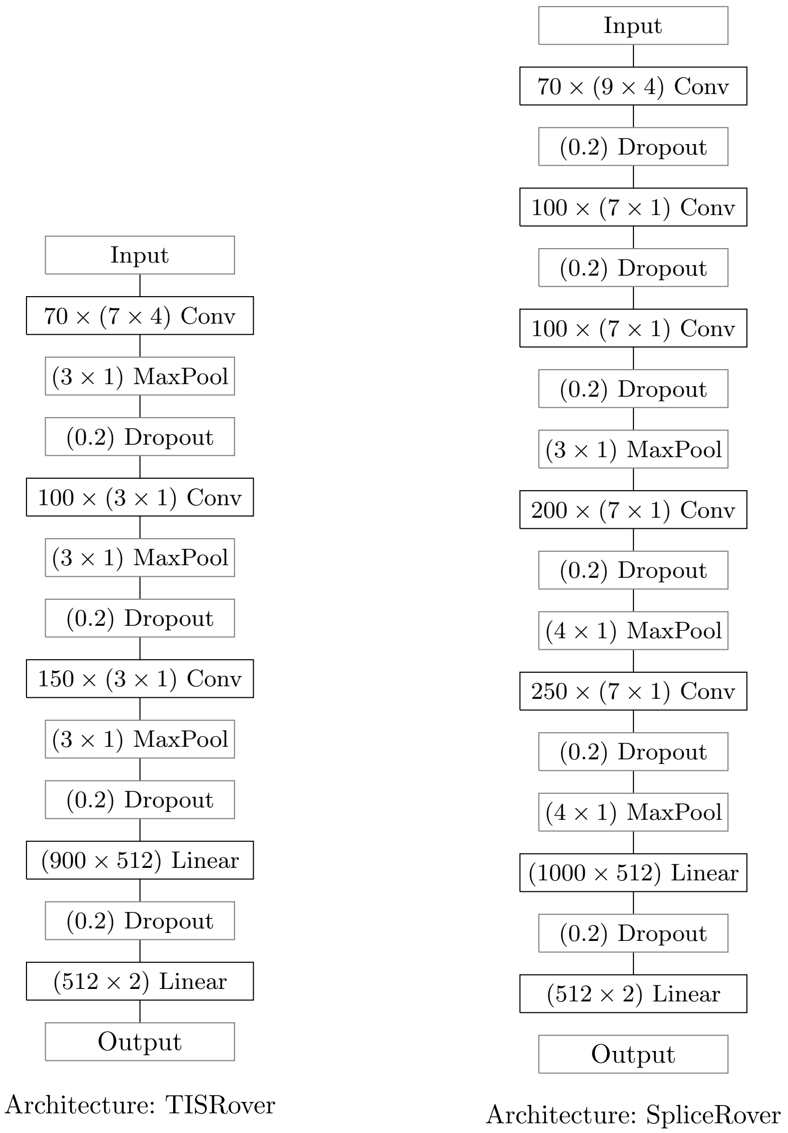

Fig. 1: Detailed descriptions for TISRover and SpliceRover architectures.

(2) early-stopping, where we stop the training if the performance on the validation set (measured with different metric for each dataset) does not increase for five consecutive epochs. The rest of the hyperparameters can be found in Table 1.

Table 1: Hyperparameters utilized during the model training process.

Hyperparameter	TISRover	SpliceRover
Warmup	1 epoch	1 epoch
Training epochs	50 epochs	100 epochs
Initial Learning Rate	0.05	0.05
Learning Rate Decay	Step decay 0.75 every 20 epochs	Step decay 0.5 every 20 epochs
Batch Size	64	64
Weight decay	0	0
Optimizer	SGD	SGD
Momentum	0.9	0.9

Table 2: Best-case results obtained with the augmentations proposed in our work for each dataset compared to the reference performance.

Dataset	Metric	Reference	Mutation			
			Silent	Mis-sense	Non-sense	Random
`Chrom21`	↓ fpr80	0.031	**0.023**	**0.022**	**0.030**	**0.023**
`Gao15`	↑ auROC	**0.866**	0.863	0.864	0.860	0.856
	↑ auPRC	0.589	0.585	**0.593**	0.587	0.581
`NN269`	↑ auROC	0.989	**0.993**	**0.994**	**0.992**	**0.993**
`A.Acc`	↑ Pr95	0.939	**0.962**	**0.971**	**0.969**	**0.962**
`A.Don`	↑ Pr95	0.956	**0.973**	0.933	**0.973**	**0.965**

2 Additional results

In Table 2, we provide a comparison of the best-performing model created with various types of mutation augmentations to the reference performance on that dataset. As it can be seen, using various types of mutations as augmentations increases performance for all scenarios except one.

Supplementary Figures 2, 3, and 4 show the distributions of accuracies across 6 repeats of the experiments, with the 3 best (top-3) accuracy values shown for each run. Each figure pane shows one particular mutation type, with the effect of up to 3 mutations. On each pane, the horizontal gray line indicates the mean of the baseline data, where no mutations are applied. An arrow underneath a distribution indicates that there is a significant difference between that population and the baseline, as determined by a non-parametric Mann-Whitney U test at the 5% significance level, with the arrow direction indicating whether this is an increase or decrease.

The p-values for these tests are described in Supplementary Tables 3 and 4, together with the differences in mean between the baseline (no mutations applied) and a specific mutation type and count (in parentheses).

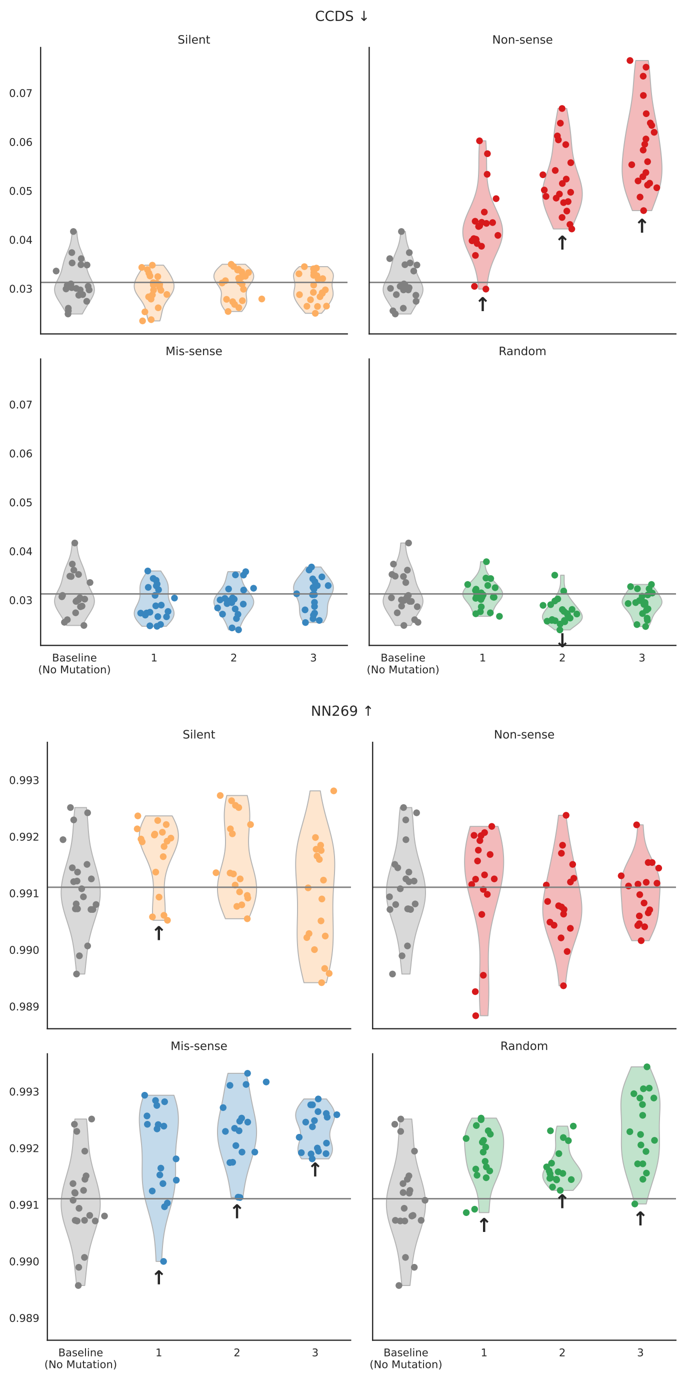

Fig. 2: Comparison between baseline accuracy and accuracy for different mutation types, for the `Chromosome-21` (top) and `NN269` (bottom) datasets. `Chromosome-21` uses fpr80 as an accuracy metric (lower is better). `NN269` uses auROC (higher is better).

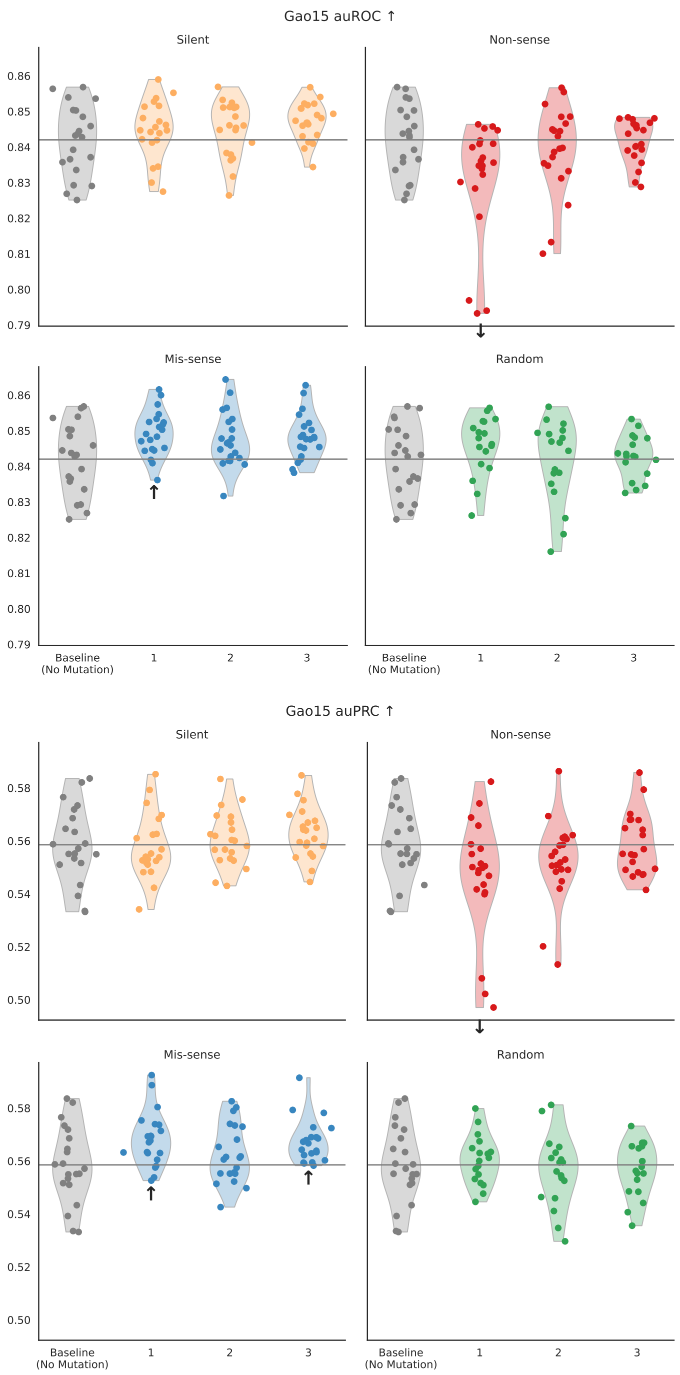

Fig. 3: Comparison between baseline accuracy and accuracy for different mutation types, for the `Gao15` dataset, using the auROC metric (top) and auPRC (bottom). For both, higher is better.

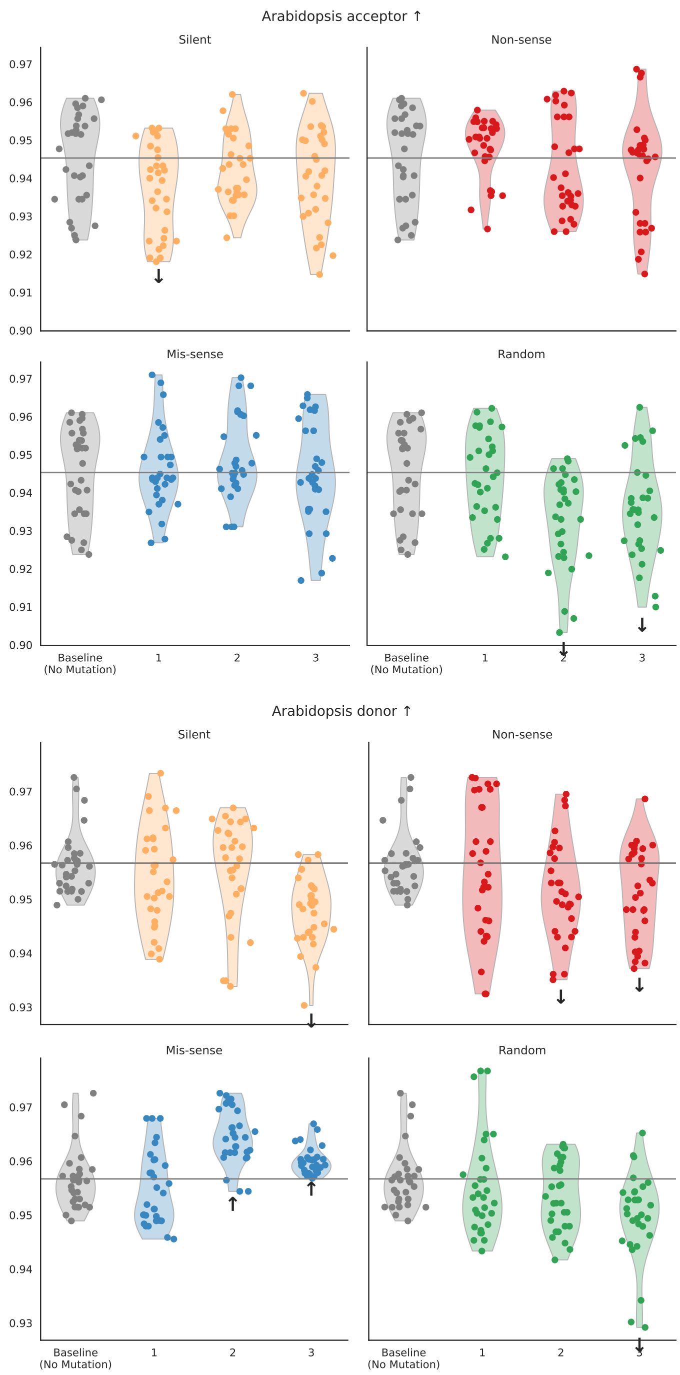

Fig. 4: Comparison between baseline accuracy and accuracy for different mutation types, for the `Arabidopsis acceptor` (top) and `Arabidopsis donor` (bottom) datasets. Both datasets use the Pr95 accuracy metric (higher is better).

Table 3: Silent and non-sense mutations: differences between mean of mutated data and baseline (no mutations). Between parentheses: p-value for Mann-Whitney-U comparison. An up/down arrow (↑/↓) indicates that the mean of mutated data increased/decreased and the change is significant.

	Silent			Non-sense		
Mut. count:	1	2	3	1	2	3
Chromosome-21	-1.4e-3 (0.40)	-5.6e-4 (0.93)	-1.0e-3 (0.46)	0.01 ↑ (2.2e-6)	0.02 ↑ (3.1e-8)	0.02 ↑ (3.1e-8)
NN269	5.8e-4 ↑ (0.03)	4.6e-4 (0.08)	-1.8e-4 (0.66)	3.5e-5 (0.48)	-2.3e-4 (0.2)	-1.1e-4 (0.50)
Gao15-auROC	2.7e-3 (0.3)	2.8e-3 (0.26)	5.1e-3 (0.07)	-0.01↓ (0.02)	-2.9e-3 (0.59)	-3.1e-4 (0.91)
Gao15-auPRC	-6.1e-4 (0.68)	1.9e-3 (0.65)	4.5e-3 (0.20)	-1.1e-2 ↓ (0.04)	-6.0e-3 (0.18)	1.8e-4 (0.78)
A.Acc	-8.2e-3 ↓ (6e-3)	-3.0e-3 (0.23)	-5.0e-3 (0.11)	2.4e-3 (0.73)	-2.2e-3 (0.55)	-2.8e-3 (0.22)
A.Don	-2.2e-3 (0.29)	-1.5e-3 (0.70)	-8.7e-3 ↓ (3e-6)	-1.7e-3 (0.60)	-5.2e-3 ↓ (0.01)	-5.4e-3 ↓ (0.04)

Table 4: Mis-sense and random mutations: differences between mean of mutated data and baseline (no mutations). Between parentheses: p-value for Mann-Whitney-U comparison. An up/down arrow (↑/↓) indicates that the mean of mutated data increased/decreased and the change is significant.

	Mis-sense			Random		
Mut. count:	1	2	3	1	2	3
Chromosome-21	-1.7e-3 (0.16)	-1.4e-3 (0.29)	-2.8e-4 (0.97)	-1.7e-4 (0.72)	-3.6e-3 ↓ (2.5e-3)	-1.9e-3 (0.13)
NN269	8.1e-4 ↑ (2.3e-3)	1.2e-3 ↑ (7.1e-5)	1.2e-3 ↑ (1.1e-5)	7.7e-4 ↑ (1.1e-3)	5.8e-4 ↑ (2.2e-3)	1.2e-3 ↑ (5.0e-5)
Gao15-auROC	7.2e-3 ↑ (0.01)	5.5e-3 (0.10)	6.3e-3 (0.05)	3.7e-3 (0.23)	-7.9e-4 (0.96)	-6.4e-5 (0.94)
Gao15-auPRC	9.7e-3 ↑ (0.02)	4.1e-3 (0.30)	8.8e-3 ↑ (0.01)	1.7e-3 (0.74)	-2.4e-3 (0.72)	-1.9e-3 (0.85)
A.Acc	7.5e-4 (0.98)	3.5e-3 (0.30)	-6.0e-4 (0.87)	-1.1e-3 (0.69)	-1.2e-2 ↓ (3.5e-4)	-9.4e-3 ↓ (8.4e-3)
A.Don	-1.8e-3 (0.14)	7.8e-3 ↑ (3.0e-6)	3.2e-3 ↑ (9.5e-4)	-1.4e-3 (0.13)	-2.9e-3 (0.16)	-6.9e-3 ↓ (3.1e-4)

References

[1] Zuallaert J, Kim M, Soete A, Saeys Y, Neve WD. TISRover: ConvNets learn biologically relevant features for effective translation initiation site prediction. International Journal of Data Mining and Bioinformatics. 2018;20(3):267–284.

[2] Zuallaert J, Godin F, Kim M, Soete A, Saeys Y, De Neve W. SpliceRover: interpretable convolutional neural networks for improved splice site prediction. Bioinformatics. 2018;34(24):4180–4188.
